# *Lactococcus lactis* engineered to deliver hCAP18 cDNA alleviates DNBS-induced colitis in C57BL/6 mice by promoting IL17A and IL10 cytokine expression

**DOI:** 10.1038/s41598-022-19455-3

**Published:** 2022-09-19

**Authors:** Esther Borras Noguès, Camille Kropp, Laureline Bétemps, Cassiana de Sousa, Florian Chain, Sandrine Auger, Vasco Azevedo, Philippe Langella, Jean-Marc Chatel

**Affiliations:** 1 Université Paris Saclay, INRAE, AgroParisTech, UMR1319, MICALIS, 78352 Jouy en Josas, France; 2 Institute of Biological Sciences, Federal University of Minas Gerais, Belo-Horizonte, MG Brazil

**Keywords:** Antimicrobials, Applied microbiology

## Abstract

With its antimicrobial and immunomodulating properties, the cathelicidin (LL37) plays an important role in innate immune system. Here, we attempted to alleviate chemically induced colitis using a lactococci strain that either directly expressed the precursor to LL37, hCAP18 (LL-pSEC:hCAP18), or delivered hCAP18 cDNA to host cells under the control of the cytomegalovirus promoter (LL-Probi-H1:hCAP18). We also investigated whether the alleviation of symptoms could be explained through modification of the gut microbiota by hCAP18. Mice were administered daily doses of LL-pSEC:hCAP18 or LL-Probi-H1:hCAP18. On day 7, colitis was induced by DNBS. During autopsy, we assessed macroscopic tissue damage in the colon and collected tissue samples for the characterization of inflammation markers and histological analysis. Feces were collected at day 7 for 16S DNA sequencing. We also performed a fecal transplant experiment in which mice underwent colon washing and received feces from *Lactococcus lactis*-treated mice before DNBS-colitis induction. Treatment with LL-Probi-H1:hCAP18 reduced the severity of colitis symptoms. The protective effects were accompanied by increased levels of IL17A and IL10 in mesenteric lymph node cells. *L. lactis* administration altered the abundance of *Lachnospiraceae* and *Muribaculaceae*. However, fecal transplant from *L. lactis*-treated mice did not improve DNBS-induced symptoms in recipient mice.

## Introduction

Inflammatory bowel diseases (IBD) such as Crohn’s disease (CD) and ulcerative colitis (UC) are multifactorial diseases associated with chronic inflammation of the gastrointestinal tract and dysbiosis of the patient’s gut microbiota^1,2^. IBD pathogenesis is characterized by excessive and uncontrolled immune responses against commensal gut bacteria^3^. While antibiotics and anti-inflammatory drugs can help to make the condition manageable, IBD can have serious complications and no curative treatment currently exists.

The innate immune system plays an important role in initiating the inflammatory response. Antimicrobial peptides (AMPs)—a broad category of immune molecules that includes defensins and cathelicidins, among others—have various modes of action^4^; they may function as wide-spectrum antibiotics that use a variety of methods to kill bacteria and fungi directly^5,6^ or act as immunomodulators by altering host gene expression, inducing chemokine production^7^, or modulating the response of the adaptive immune system^8^. AMPs have a crucial role in host defense against pathogens and the regulation of inflammatory responses by immune cells. Many AMPs are typically expressed in response to gut infection and inflammation, and the administration of some of them improves colitis symptoms. HNP1-3, secreted by neutrophils, are increased in active IBD mucosa^9^. Low doses of HNP-1 have a protective effect on DSS-induced colitis without affecting cytokines expression levels^10^. HD5 is also increased in the colon of IBD patients and has protective effect on DSS-induced colitis^9^. On the contrary, the expression of cathelicidin is reduced in UC patients and its lowered circulation level is associated with poor prognosis^11^. AMPs are currently being investigated as an alternative to antibiotics to prevent the spread of multiple-drug-resistant bacteria^12,13^.

In the gut, the innate immune response and the production of AMPs play an essential role in maintaining homeostasis. In particular, much attention has focused on the family of AMPs known as cathelicidins, which are found in both mammals and non-mammals^14,15^. These peptides have been shown to influence inflammation and wound healing^16–18^, mediate changes in the gut microbiota, and protect mice against chemically induced colitis. He have seen earlier that LL37 plays an important role in UC patients, who demonstrate a decreased circulating level of this molecule^11,19,20^. Human cathelicidin is encoded by the *CAMP* gene, the translation of which yields hCAP18, a small 18-kDa peptide. Following exocytosis, its C-terminal region is cleaved via the action of neutrophil proteinase 3 to form the 4.5-kDa peptide LL37^21^. LL37 is able to disrupt bacterial membranes and thus has direct antibacterial properties^22^. It has been found in neutrophil extracellular traps and is also produced at the surface of various epithelia where it helps fight against pathogens^23–25^. Moreover, studies have described the immunomodulating and -stimulating effects of LL37 in diverse inflammation-related settings such as epithelial cell activation, chemotaxis, angiogenesis, and tissue wound repair. In particular, LL37 has been shown to use the Fprl1 receptor to mobilize human leucocytes, neutrophils, and T cells^26^. It is also able to suppress collagen synthesis, as the administration of exogenous cathelicidin in mice and human colonic fibroblasts has antifibrogenic effects^27,28^.

Multiple studies suggest that the composition of the gut microbiota—which can be modulated by the environment^29,30^, diet^31,32^, or drug treatment^33^—influences colitis severity. In humans, IBD is clearly associated with gut microbiota alterations although it is still unclear if the effect is causal^1^. One study found that mice who were deficient in mCRAMP (the hCAP18 ortholog in mice) were highly sensitive to DSS-induced colitis, which could be prevented by mCRAMP administration. Alterations were also evident in the gut microbiota of these mice, with increased levels of bacteria from the oral microbiota in their feces^34^. In the same study, wild-type mice that were co-housed with mCRAMP-deficient mice became highly sensitive to DSS-induced colitis, suggesting a causal link between gut microbiota and disease severity. In 2013, Divyendu Singh et al. found that administration of LL37 or mCRAMP could both inhibit LPS-induced IL6 production in human and mouse cell lines. Furthermore, LL37 could up-regulate dsRNA-induced innate response through TLR3 but not mCRAMP^35^. LL37 can also inhibit LPS-induced pyroptosis of macrophages, modulate the expression of pro-inflammatory cytokines and improve survival in septic mice^36^. Recently, a Cecropin-LL37 hybrid peptide was constructed and found to be promising in the treatment of EHEC infections in a mouse model by modulating gut microbiota, inflammation and mucosal barrier^37^.

Therapies based on the oral administration of AMPs face significant challenges, as these molecules are quickly degraded before reaching the target site. For this reason, effective doses are typically very high, which raises problems associated with the high production costs and an increased potential for toxic side effects. To more efficiently and cost-effectively produce and deliver AMPs to their target site, one novel strategy is the use of recombinant probiotics^38,39^. The concept of probiotics was first introduced by Lilly and Stilwell in 1965^40^; they are live microorganisms that are considered safe for human consumption, with health benefits provided by their interactions with the gut microbiota or the host intestinal epithelium. Health benefits aside, probiotics have been proposed as a novel mechanism for drug delivery because of their ability to transit and interact with the intestinal barrier and gut bacteria^41^. Specifically, lactic acid bacteria, and *Lactococcus lactis* in particular, have attracted attention as a result of their health benefits, their lack of toxic metabolites, and their low number of secreted proteins. To date, these strains have been engineered to serve as vehicles for the production of therapeutic molecules and the delivery of DNA vaccines^41–44^. *L. lactis* that has been manipulated to contain a eukaryotic expression cassette containing cDNA of a protein of interest can be used as a delivery system for DNA that can then be expressed by the host’s cells after transfection^45–47^.

Here, we administered a recombinant strain of *L. lactis* that expresses hCAP18 (LL-pSEC:HCAP18) to mice and then induced colitis in these mice through DNBS intrarectal injection. Additionally, we used *L. lactis* as a vehicle for the delivery of hCAP18 cDNA (LL-Probi-H1:hCAP18) to the gut epithelium of mice. This second strategy allowed the direct expression of the peptide by the host’s cells^48^. We collected feces at day 0 and day 7 after bacterial administration for microbiota sequencing and fecal transplant into other groups of mice. Our aims were to compare the two strategies of AMP delivery with respect to their effectiveness in alleviating chemically induced colitis in mice, to understand the impact of each strategy on the host microbiota, and to determine if the mode of action of hCAP18 on the disease is dependent on its modulation of the gut ecosystem of the host.

## Results

### Delivery of hCAP18 cDNA alleviates DNBS-induced symptoms

**Figure 1:**
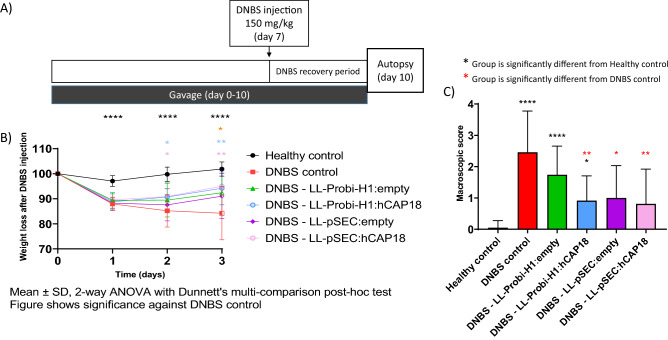
Effect of DNBS injection on weight loss and macroscopic score on experimental groups receiving recombinant *L. lactis* or not. (**A**) Experimental protocol for the DNBS-induced model of colitis in mice. Effects of LL-Probi-H1-empty, LL-Probi-H1-hCAP18, LL-pSEC:empty and LL-pSEC:hCAP18 on recovery from DNBS with respect to (**B**) body weight and (**C**) macroscopic score. Data represent mean ± SD. *P < 0.05, **P < 0.01, ***P < 0.001, ****P < 0.0001, n = 24 mice per group for LL-Probi-H1:empty and LL-Probi-H1:hCAP18, n = 16 for LL-pSEC:empty and LL-pSEC:hCAP18, n = 20 mice for healthy control.

**Figure 2:**
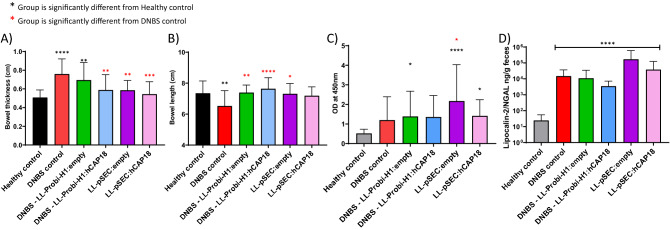
Effects of preventive treatment on DNBS-induced tissue damage and inflammation. Assessments of (**A**) bowel thickness, (**B**) bowel length, (**C**) MPO activity, and (**D**) lipocalin in feces in control groups (PBS-glycerol, DNBS) and treatment groups (LL-Probi-H1:empty, LL-Probi-H1:hCAP18, LL-pSEC:empty, LL-pSEC:hCAP18). Data represent mean ± SD. *P < 0.05, **P < 0.01, ***P < 0.001, ****P < 0.0001, n = n = 24 mice per group for LL-Probi-H1:empty and LL-Probi-H1:hCAP18, n = 16 for LL-pSEC:empty and LL-pSEC:hCAP18, n = 20 mice for healthy control.

The functionality of the ProBiH1:hCAP18 vector was verified by Western blot; a specific band at 18 kDa was detected in protein extracts 24 h after transfection into HEK293 cells (data not shown). We then assessed the effects of the LL-ProBi-H1:empty or LL-Probi-H1:hCAP18 recombinant strains following 7 days of gavage in a mouse model of acute DNBS-induced colitis (Fig. 1A). One day after DNBS injection, all groups of mice (DNBS control, LL-ProBi-H1:empty, and LL-Probi-H1:hCAP18) had lost 10% of their body weight; three days after injection, mice in the DNBS control group had lost 15% of their weight and did not show any signs of recovery. Instead, mice that received treatment with either recombinant strain stabilized their weight after day 1 and began to recover some of the lost weight (Fig. 1B). Macroscopic scores measured 3 days after DNBS injection were significantly better (2.7-fold) in mice that received LL-Probi-H1:hCAP18 compared to the DNBS control group (Fig. 1C), while mice that were treated with LL-ProBi-H1:empty showed no statistically significant improvement. Similarly, DNBS injection caused significant thickening of the bowel wall (0.76 mm), which was alleviated by LL-Probi-H1:hCAP18 (0.59 mm) but not LL-ProBi-H1:empty (Fig. 2A). Bowel length was likewise affected by DNBS injection (7.34 cm in healthy control versus 6.52 cm in DNBS control) and this effect was alleviated by both LL-ProBi-H1:empty and LL-Probi-H1:hCAP18.

To evaluate the effects of LL-Probi-H1:hCAP18 on neutrophil infiltration, we measured the activity of myeloperoxidase (MPO) and the expression of lipocalin-2 in feces (Fig. 2C,D) DNBS injection induced a significant increase of MPO activity in LL-Probi-H1:empty, LL-pSEC:empty and LL-pSEC:hCAP18 groups. DNBS induced MPO activity of LL-pSEC:empty treated mice was significantly higher than non-treated control mice. Compared to healthy controls, concentrations of lipocalin-2 in feces were much higher in all groups that received DNBS injections; however, the group treated with LL-Probi-H1:hCAP18 had significantly lower lipocalin-2 expression than the DNBS control group (Fig. 2D).

### LL-pSEC:hCAP18 does not significantly improve DNBS-induced symptoms compared to LL-pSEC:empty

The functionality of the pSEC:hCAP18 plasmid was verified by Western blot following nisin induction of the LL-pSEC:hCAP18 strain. A specific band at 18 kDa was detected (data not shown). We then used this strain to study how the administration of hCAP18 in protein form differed from the effects of cDNA. Weight recovery was not different between LL-pSEC:empty and LL-pSEC:hCAP18 mice (Fig. 1B). Both LL-pSEC:hCAP18 and LL-pSEC:empty improved macroscopic score and reduced colon thickness compared to DNBS control (Figs. 1C, 2A). There were no significant differences among groups with respect to lipocalin-2 concentration in feces and MPO activity (Fig. 2C,D).

### Delivery of hCAP18 does not alleviates DSS-induced symptoms

**Figure 3:**
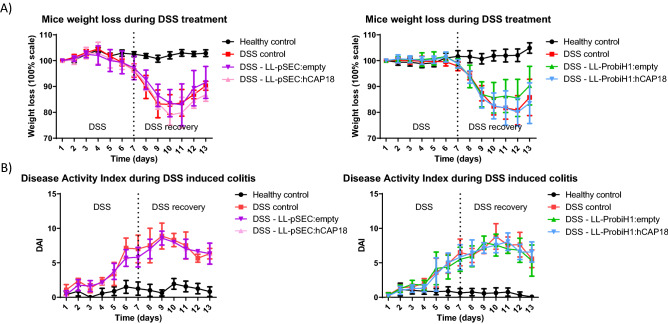
Effect of DSS on weight loss and disease activity index (DAI)on experimental groups receiving recombinant *L. lactis* or not. Effects of LL-Probi-H1-empty, LL-Probi-H1-hCAP18, LL-pSEC:empty and LL-pSEC:hCAP18 on recovery from DSS with respect to (**A**) body weight and (**B**) Disease Activity Index. Data represent mean ± SD. n = 8 mice per group.

Mice were given DSS in drinking water for 6 consecutive days (1 day = 24 h cycle) then sacrificed 12 days after the beginning of DSS administration. We found no significant difference in the weight loss and Disease Activity Index (calculated with the consistency and presence of blood in feces) in our mice (Fig. 3). No further analysis was performed after this experiment.

### Probi-H1:hCAP18 increases local IL10, IL17A production and reduces IFN-γ secretion

**Figure 4:**
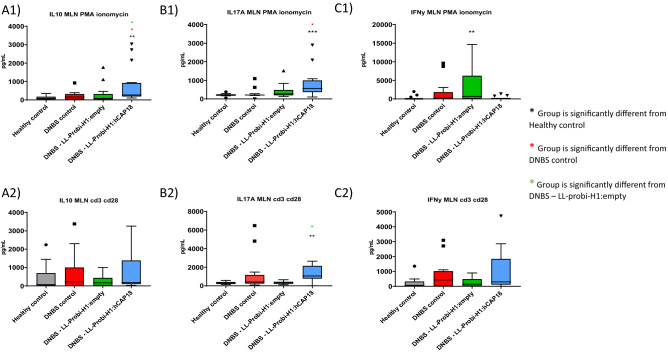
Cytokine concentrations in mesenteric lymph node supernatants. Cytokine secretion by mesenteric lymph node cells was induced with PMA ionomycin (**A1**, **B1**, **C1**) or CD3+/CD28+ (**A2**, **B2**, **C2**), A = IL-10, B = IL-17A, C = IFN-γ. Box and whiskers were plotted using the Tukey method. Statistical significance was determined by Kruskal–Wallis with Dunn’s multiple comparison test. *P < 0.05, **P < 0.01, ***P < 0.001, ****P < 0.0001, n = 16 mice per group, points shown on graph represent outliers outside of range.

We studied the effects of LL-Probi-H1:hCAP18 on the local immune response by measuring cytokine production in mesenteric lymph node (MLN) cells from our mice following stimulation with PMA ionomycin or CD3+/CD28+. We observed significantly higher levels of the anti-inflammatory cytokine IL-10 in the MLN cell supernatant of the LL-Probi-H1:hCAP18 group compared to the DNBS and LL-Probi-H1:empty groups following stimulation with PMA ionomycin (Fig. 4A1,A2). Furthermore, following reactivation with either method, IL17A production was high in the MLN cell supernatants from the LL-Probi-H1:hCAP18 group, suggesting an increased Th17 effector cell response (Fig. 4B1,B2). Finally, the PMA ionomycin–treated MLN cell supernatants from the LL-Probi-H1:empty group demonstrated high levels of the pro-inflammatory cytokine IFN-γ (Fig. 4C1,C2).

We also studied the expression of TNF-α, IL17A, and IL22 mRNA in colon tissues and found no significant difference between our treatment groups (Fig. S1).

### Comparison of fecal microbiota composition among treatment groups

**Figure 5:**
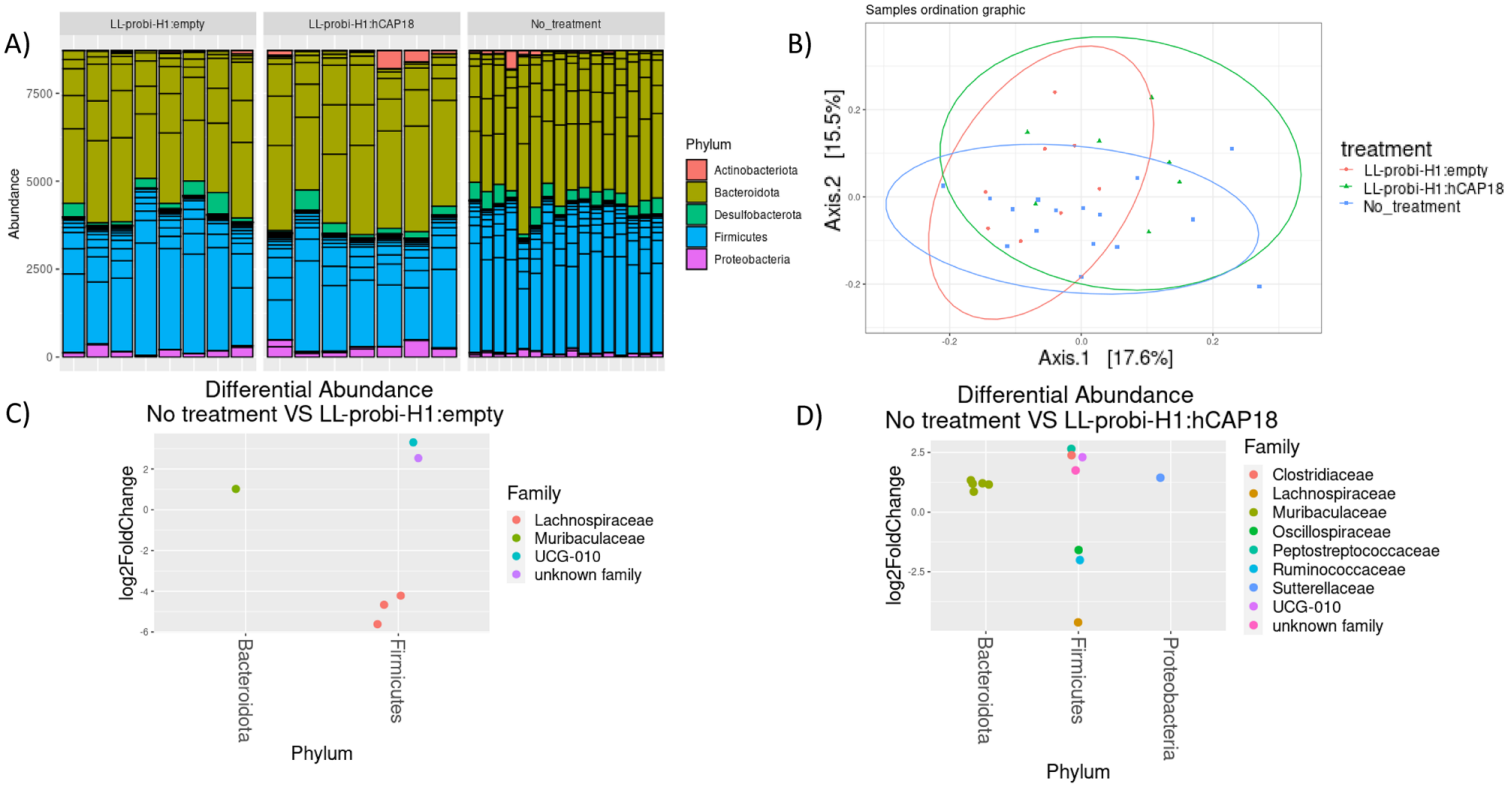
Analysis of the composition of fecal microbiota by 16S sequencing after 7 days of gavage. (**A**) Composition of samples by phylum. (**B**) Principal coordinates analysis of different treatment groups. (**C**) Differentially abundant taxa between untreated and LL-probi-H1:hCAP18 groups and (**D**) untreated and LL-probi-H1:empty groups, according to DESeq2. Untreated control group n = 16, LL-Probi-H1:hCAP18 n = 7, LL-Probi-H1:empty n = 8.

We analyzed fecal microbiota composition by 16S sequencing after 7 days of gavage with PBS glycerol and LL-ProBiH1:empty or LL-ProBiH1:hCAP18. No significant difference in alpha diversity was found among groups (observed or Shannon index), although mice that received recombinant *L. lactis* had slightly decreased diversity compared with the PBS glycerol control group. With respect to the composition of the fecal microbiota, we detected a slight shift in mice that received LL-Probi-H1:empty or LL-Probi-H1:hCAP18 compared to control mice; *L. lactis* treatment was found to be responsible for a portion of the variation in the data (PERMANOVA, R^2^ = 0.114537, F = 1.8079, p = 0.005, see Fig. 5B, Table S1). However, there was no relationship between patterns of community composition and macroscopic score (PERMANOVA, R^2^ = 0.16878, F = 1.0152, p = 0.424, see Table S2). We examined a total of 704 OTUs in 31 total samples; these represented 5 phyla (Fig. 5A), 8 classes, 23 orders, 33 families, and 77 identified genera. Patterns of differential abundance were evaluated using the DESeq2 package in R. Compared to the control PBS glycerol group, several taxa were differentially abundant in the LL-Probi-H1:hCAP18 group (9 families, 13 OTUs) and LL-Probi-H1:empty group (4 families, 6 OTUs). In the former comparison, the taxa affected were identified as Muribaculaceae (5 OTUs), Ruminococcaceae (2 OTUs), and Lachnospiraceae (1 OTU) (Fig. 5D, Table S3), while the taxa highlighted in the latter comparison were assigned to Muribaculaceae (1 OTU), Ruminococcaceae (1 OTU), Clostridia (1 OTU), and Lachnospiraceae (3 OTUs) (Fig. 5C). However, only 1 OTU, from the Muribaculaceae family (cluster_116), was highlighted by both contrasts (Table S3). Instead, a comparison of the LL-Probi-H1:empty and LL-Probi-H1:hCAP18 groups revealed no patterns of differential abundance.

### Effects on DNBS-induced colitis of fecal transplant from mice treated with LL-ProBi-H1:empty or LL-ProBi-H1:hCAP18

**Figure 6:**
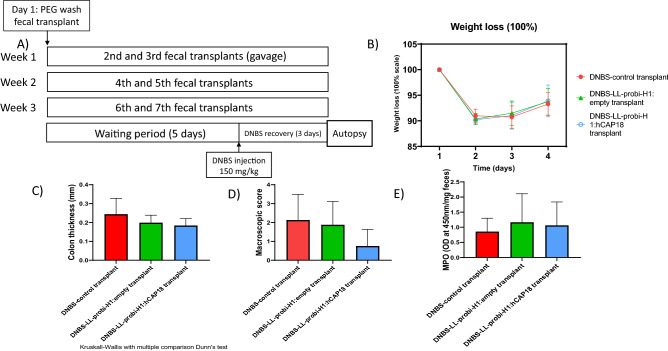
Effect on DNBS-induced colitis of fecal transplant from donor mice that were gavaged with LL-Probi-H1:empty or LL-Probi-H1:hCAP18. (**A**) Experimental protocol of PEG washing and fecal transplant. (**B**) Mouse weight loss. (**C**) Colon thickness. (**D**) Macroscopic score. (**E**) MPO activity. Data represent mean ± SD. *P < 0.05, **P < 0.01, ***P < 0.001, ****P < 0.0001, n = 8 mice per group.

In order to evaluate whether, and to what extent, the protective effects we detected were mediated by shifts in the microbiota, we performed fecal transplants from donor mice who were gavaged with PBS glycerol and either LL-ProBi-H1:empty or LL-ProBi-H1:hCAP18. Recipient mice were first subjected to colon washes with PEG and then received oral gavages of fecal material from donor mice following the protocol shown in Fig. 6A. After transplantation, the recipient mice were injected with DNBS in order to induce colitis. There were no significant differences between control and treatment groups with respect to symptoms such as weight loss (Fig. 6B), colon thickness (Fig. 6C) or macroscopic score (Fig. 6D). There were also no differences in MPO activity (Fig. 6E) among the groups.

## Discussion

In our study, we investigated the protective effect of human cathelicidin (hCAP18) in DNBS induced colitis, using *L. lactis* as a vector for the delivery of either the protein or cDNA forms of this molecule. *L. lactis* is a food-grade lactic acid bacterium that is considered to be non-invasive and non-colonizing, and has previously demonstrated potential for this type of treatment. For example, after 7 days of DSS-induced colitis in mice, the administration of 10 log cfu of *L. lactis* that was engineered to express mCRAMP under the control of nisin was found to reduce clinical symptoms and tissue damage better than sulfasalazine^49^ and was associated with lower expression of TNF-α and IL-1β. Another team found similar results with reduction of pro-inflammatory cytokines IL-6, IL-1β, TNF-α, increased production of IL-10 and upregulation of tight junction proteins ZO-1, ZO-2 and occludin^50^. Here, we observed that treatment with the recombinant strain LL-Probi-H1:hCAP18 reduced DNBS-induced colitis symptoms in mice. Interestingly, when we repeated the experiment in a DSS-induced model of colitis we found no significant effect on symptoms induced by DSS (Fig. 3). The discrepancy between the results of our DSS experiment and this previous work may be explained by the different protocols used: unlike the earlier study, we included a recovery period of 5 days after DSS treatment and used a cathelicidin of human origin. However, DSS acts as a toxin against the gut epithelium and induces injury^51^ whereas ethanol-induced permeability of the gut epithelium will allow DNBS to penetrate the colonic tissues and trigger the host’s innate and adaptive immune responses^52^. This difference in mechanism could partly explain why hCAP18 is inefficient in DSS-induced colitis. Further investigation is needed to fully address this issue.

One of our main goals was to compare how the effects of hCAP18 differ depending on its mode of delivery: exogenous delivery (LL-pSEC:hCAP18) or endogenous production after delivery via DNA vaccine (LL-Probi-H1:hCAP18). We first verified that the LL-pSEC:hCAP18 strain was able to produce hCAP18 and that Probi-H1:hCAP18 could induce hCAP18 production in mammal cells using Western blot analysis (Fig. S2). We then characterized the effects of both strains in mice with DNBS-induced colitis. Treatment with LL-Probi-H1:hCAP18 improved disease symptoms compared to DNBS control (see Fig. 1 and 2; weight recovery, bowel length, bowel thickness, and macroscopic tissue damage). The administration of LL-Probi-H1:empty also had a positive effect on weight recovery and bowel length; however, compared to healthy controls, expression of the pro-inflammatory cytokine IFN-γ was significantly higher in the MLN cells of mice in this group. Treatment with LL-Probi-H1:hCAP18 restored IFN-γ to normal levels (Fig. 4C1). Symptoms in the mice treated with LL-pSEC:empty or LL-pSEC:hCAP18 were also significantly ameliorated compared to the DNBS control group, but there was no difference between these two treatment groups (Figs. 1 and 2). It thus appeared that *L. lactis* by itself had protective properties toward DNBS-induced symptoms that could mask the effects of the hCAP18 it produced. This was unexpected as our previous experiments with this *L. lactis* strain found this bacterium could exacerbate DNBS-induced colitis weight loss and had no positive effects on the macroscopic score^53^. However, a multitude of *L. lactis* strains have protective effects in colitis models^54–56^ and it is possible that yet unidentified variables played a role in the exacerbation of DNBS-induced colitis previously observed. We note that despite the decrease in weight loss and tissue damage, *L. lactis* still increased the production of the pro-inflammatory cytokine IFN-γ, which is consistent with our previous findings^57^. Additionally, we observed that endogenous production of hCAP18, as induced by DNA vaccine delivery, strengthened the protective effects of this bacterium; mice treated with LL-Probi-H1:hCAP18 were more protected from developing symptoms of colitis than those who were treated with LL-pSEC:hCAP18. This result may be due to differences in the final processing step of hCAP18. As noted earlier, in order to produce the antimicrobial peptide LL37, hCAP18 must be processed by proteinase 3, which is mainly produced and secreted by neutrophils^13^. We can hypothesize that hCAP18 delivered into the lumen by LL-pSEC:hCAP18 is less accessible to proteinase 3 than hCAP18 produced by epithelial cells after delivery by LL-Probi-H1:hCAP18. Although this point remains to be clarified, our results confirm the potential of *L. lactis* as a vehicle for the production of therapeutic molecules via DNA vaccine delivery.

Treatment with LL-Probi-H1:hCAP18 was also associated with elevated levels of the anti-inflammatory cytokine IL10. IL10 is an important regulator of intestinal homeostasis and prevents pro-inflammatory responses by T-cells^58^ and macrophages^59^. Surprisingly, we also found increased levels of putatively proinflammatory IL17A in MLNs of this group. High levels of this cytokine have been found in patients with an active form of ulcerative colitis^60^, but the beneficial or detrimental nature of its effects are still up for debate. IL17 KO mice subjected to DSS-induced colitis show increased survival and reduced tissue damage compared to wild-type controls^58^. Anti-IL17 treatments such as Ixekizumab, Brodalumab, or Secukinumab are FDA-approved and widely used in the treatment of psoriasis^61^ but, paradoxically, the use of IL17 blockers to treat IBD has been shown to exacerbate symptoms^62–64^. Similarly, the use of anti-IL17 monoclonal antibodies was found to exacerbate DSS-induced colitis in mice and increase the expression of pro-inflammatory markers such as TNF-α, IFN-γ, and IL-6^65^. Other research has reported an association in colitis between IL17 and preservation of epithelial barrier integrity through stimulation of cell proliferation and AMP production^66^. A 2020 study suggested that IL17A deficiency in chronic colitis upregulates IL6 expression and leads to the recruitment of RORγt + innate lymphoid cells in a negative feedback loop; the authors proposed this mechanism to explain the discrepancies between the insensitivity of IL17A-KO mice to acute colitis and the otherwise negative correlation between IL17A and worsening symptoms^67^. However, increased IL17A levels are not necessarily associated with worse symptoms in all models of acute colitis; its effects are heavily dependent on the surrounding circumstances. For example, IL10^−/−^ mice spontaneously develop colitis that is greatly aggravated by IL17A deficiency^68^, while Minns et al. found that mCRAMP is required for Th17 differentiation and reported increased production of IL17A in the context of inflammation^69^.

In order to investigate the importance of the anti-microbial activity of hCAP18 in the protective effects noted here, we studied how an increase in host production of hCAP18, and potentially LL37, affected the composition of the gut microbiota. Previous findings suggested that mCRAMP deficiency modified the microbiota in such a way as to make wild-type cage mates of mCRAMP^−/−^ mice more susceptible to DSS-induced colitis^34^. We characterized the microbiota of mice using 16S sequencing of fecal samples and observed a slight but significant shift in community composition in LL-Probi-H1:hCAP18-treated mice compared to the control group. Multiple OTUs assigned to family *Muribaculaceae* increased in abundance, whereas certain members of *Lachnospiraceae* declined (Fig. 4). However, we must keep in mind that the sequencing method we used only gives us a global view of the microbiota composition, and it is difficult to determine if such minor changes are relevant. For this reason, we also tested if the microbiota from treated mice could protect untreated recipient mice from inflammation. We found that fecal transplantation from LL-Probi-H1:hCAP18–treated mice to PEG-washed recipient mice did not significantly reduce symptoms of DNBS-induced colitis (Fig. 5). One potential explanation of this result is that these wild-type mice had normal expression of mCRAMP during inflammation, and additional cathelicidin might not have changed the microbiota in a helpful way. Although, it is possible that the antimicrobial effects of hCAP18 are specific to human-associated microbiota and therefore would not have much impact on mice-associated species. To fully understand the effects of LL37 on the microbiota, further investigation should be done on a local scale by analyzing the composition of the mucosal microbiota.

## Materials and methods

### Plasmid cloning and *L. lactis* transformation

hCAP18 synthetic cDNA optimized for *L. lactis* expression was cloned into a pSEC plasmid as described in^70^ to create pSEC:hCAP18; this was then transformed in *L. lactis* NZ9000 to create the recombinant strain LL-pSEC:hCAP18, in which the induction of hCAP18 was nisin-dependent. hCAP18 synthetic cDNA optimized for expression in mice was cloned into the proBi-H1 plasmid to create proBi-H1:hCAP18; this plasmid vector contained the repA and repC origin of replication, the chloramphenicol resistance gene, and the expression cassette from pcDNA3 (Invitrogen) as published before^71^. The resulting vector was 3.7 kb long. The proBi-H1:hCAP18 plasmid was transformed in *Lactococcus lactis* MG1363 to create the recombinant strain LL-ProBi-H1:hCAP18.

### Bacterial strain preparation

LL-pSEC bacteria were preincubated at 30 °C overnight in M17 broth medium with 0.5% glucose and chloramphenicol (10 µg/mL). The optical density (OD) of the initial culture was 0.2; nisin (10 ng/mL) was added 1 h after the start of incubation and bacteria were incubated for another 1.5 h (OD = 1.2). Bacteria were harvested by centrifugation at 8000×*g* for 5 min at 4 °C and washed 3 times with PBS. They were concentrated 5 times in PBS with 16% glycerol and frozen until gavage.

LL-Probi-H1 bacteria were preincubated in the same conditions; initial culture OD was 0.2 and culturing ended at OD 1.7–1.8. Bacteria were harvested by centrifugation at 8000×*g* for 5 min at 4 °C and washed 3 times with PBS. They were concentrated 10 times in PBS with 16% glycerol and 10^9^ cfu of bacteria were administered orally to mice every day. Cell counts were quantified on M17 with 0.5% glucose (10 µg/mL of chloramphenicol) agar plates to assess viable cfu concentrations.

### HEK293 culture cell for protein expression of plasmid

Probi-H1:hCAP18 functionality was checked by transfection in HEK293 cells with Probi-H1:empty as control. The HEK293 cell line came from the American Type Culture Collection (ATCC). Cells were grown in Dulbecco’s modified Eagle’s medium + DMEM with GlutaMAX (Gibco) supplemented with 10% heat-inactivated fetal bovine serum (Eurobio Scientific), 1% sodium pyruvate (Gibco), and 1% streptomycin/penicillin (Sigma-Aldrich), and incubated at 37 °C in a humidified atmosphere with 10% CO2. Cells were passed at 80% confluence and seeded in 24-well plates at 8*10^4 cells/well, then incubated for 24 h.

Transfection of 50 ng of plasmids was performed with Lipofectamine LTX with Plus Reagent (Invitrogen) in opti-MEM medium (Gibco, Life Technologies). HEK293 cells were co-incubated with their plasmid mix for 4 h after which the medium was replaced with complete medium. After 24 h of incubation, the supernatant was removed and cells were lysed in Passive Lysis Buffer 1X (Promega). Protein expression was then checked by western blot.

### Protein extraction from *Lactococcus lactis*

To control the expression of hCAP18 by LL-pSEC:hCAP18, bacteria were incubated overnight in 10 mL of broth medium and harvested by centrifugation at 8000×*g* for 5 min at 4 °C. Bacteria were physically lysed with Precellys in cOmplete Protease Inhibitor Cocktail (Roche) and cell debris was removed by centrifugation at 8000×*g* for 5 min. Protein expression was then checked by western blot.

### Western blot

Protein extracts were boiled at 95 °C for 5 min in Laemmli buffer. Gel electrophoresis was performed with 4–20% Mini-PROTEAN TGX Precast Protein Gels (Bio-Rad) and PageRuler Prestained Protein Ladder (Thermoscientific). Proteins were then transferred on membrane using Trans-Blot Turbo Transfer Pack (Bio-Rad). Nitrocellulose membrane were saturated in tris-buffered saline, 0.1% tween, 5% dry milk. Primary antibody was the monoclonal LL-37/CAP-18, Human, mAb 3D11 (Hycult Biotech) and secondary antibody was goat-produced Anti‑Mouse IgM (µ) Affinity purified antibody HRP. The substrate mixture used was the Clarity Western ECL Substrate (Bio-Rad). Digital imaging was performed on the ChemiDoc MP System.

### Experimental conditions for mice

Male C57BL/6JRj mice (6 weeks old) were purchased from Janvier Labs (France) and kept at the INRAE animal facility in accordance with institutional ethical guidelines. The study was approved by the COMETHEA ethics committee (“Comité d’Ethique en Expérimentation Animale”) of the Centre INRAE of Jouy-en-Josas and AgroParisTech (n°16744-201807061805486). It was reported in accordance with the ARRIVE guidelines. They were fed ad libitum, given autoclaved tap water, and housed in groups of four mice per cage in an air-conditioned room with controlled temperature and circadian cycle (12 h light, 12 h dark).

### *Lactococcus lactis* administration and induction of colitis

Colitis was induced by a single intrarectal injection of DNBS (150 mg/kg of body weight) dissolved in 30% ethanol. Mice were anesthetized prior to injection. Control mice were injected with 30% ethanol. Mice were monitored daily for weight loss after intrarectal injection.

For the DNBS experiments, mice were divided into six different groups: healthy control, DNBS control, DNBS + 10^9^ cfu LL-pSEC:empty (nisin induction), DNBS + 10^9^ cfu LL-ProBiH1:empty (DNA delivery system), DNBS + 10^9^ cfu LL-pSEC:hCAP18, or DNBS + 10^9^ cfu LL-ProBiH1:hCAP18. LL-pSEC strains were tested 2 × 8 mices replicates and LL-ProBiH1 strains in 3 × 8 replicates. Bacteria were administered daily by intragastric gavage for 7 days prior to intrarectal injection of DNBS. Gavages continued until the end of the experiment, 3 days after DNBS injection. After DNBS injections, mice were monitored daily for weight loss. Mice were sacrificed by cervical dislocation at day 3 after injection, the abdomen was opened by midline incision, the colon and Mesenteric Lymphatic Node (MLN) were removed. MLN were stocked in DMEM medium on ice. They were later mashed and counted before stimulation with antiCD3 and anti-CD28 antibodies for 48 h at 37 °C and 10% CO2. Colons were opened longitudinally, cleaned and measured for scoring.

The *L. lactis* treatments on DSS-induced colitis were done in two separate experiments of 8 mice per group. We added 2% dextran sulfate sodium salt (MPBio) in the drinking water for 6 consecutive days (1 day = 24 h hours). Mice were sacrificed by cervical dislocation at day 13 after DSS induction. Mice were monitored daily for weight loss and Disease Activity Index (DAI) which was evaluated following the protocol by Cooper et al.^72^.

### PEG wash and fecal transplantation

In the fecal transplantation group, mice were fasted and the cage litter was changed 1 h before colon washing, in which mice were fed 200 µL polyethylene glycol (PEG) four consecutive times, with a 20-min interval between feedings, as described in^73^. They were then fed 200 µL fecal content by intragastric gavage 3 h after the final PEG dose. Fecal content was given to mice twice a week for 3 weeks after the initial PEG wash.

### Macroscopic scoring and histologic sampling

Macroscopic tissue damage was assessed the day of autopsy using the Wallace score^74^. Macroscopic criteria for scoring include the presence of adhesions between the colon and other organs, colon wall thickening, gastrointestinal transit issues, ulcers and hyperemia. Colon thickness was measured at the anal extremity and colon length was measured between the anal extremity and caecal junction.

### Chemokines secretion by stimulated lymphocytes

Mesenteric Lymph Nodes (MLN) were taken from mice during autopsy. They were crushed and filtered (70 µm, BD biosciences) in DMEM (Gibco). Lymphocytes were counted by flow cytometry (Accuri C6, Dutscher) and suspended in DMEM with 100 Unit of Streptomycin, Penicillin (Sigma) and 10% Fetal Calf Serum (FCS) (Eurobio) at a concentration of 2.5 × 10^5^ cells/mL either in 24 wells plate (Costar) pre-incubated with anti-CD3 and anti-CD28 antibodies, 4 µg/mL of each antibody (eBioscience) in PBS or stimulated with PMA ionomycin (eBioscience). Plates were incubated 48 h at 37 °C, 10% of CO2 and cytokine level was assessed by ELISA MAX Standard Set Mouse (BioLegend). The cytokines tested were Th1-related cytokine (IFNγ); Th17-related cytokine (IL17) and Treg– related cytokine (IL10).

### Myeloperoxidase activity in colon

Colon samples were homogenized using a Precellys Evolution (Bertin Instruments) in a hexadecyltrimethylammonium bromide buffer (Sigma-Aldrich). MPO was detected with O-dianisidine solution (Sigma-Aldrich) with 1% hydrogen peroxide solution (Sigma-Adrich) added just before measurement. OD was measured using Infinite M200 PRO (TECAN) and normalized to colon mass.

### mRNA extraction and qPCR

RNA extraction was performed with the RNeasy Mini Kit (Qiagen) according to the manufacturer’s recommendations, with the use of the RNase-Free DNase Set (Qiagen). Reverse transcription was performed with an High-Capacity cDNA Reverse Transcription Kit (Applied Biosystems). Real-Time PCR was performed with SYBR MasterMix dTTP Blue (Takyon Rox) with the primers and their source described in Table 1. Primers were synthetized by Eurofins Scientific.

**Table 1:** List of primers.

Gene	Forward	Reverse	Source
GAPDH	AACTTTGGCATTGTGGAAGG	ACACATTGGGGGTAGGAACA	Diane Nam, PLoS One, 2012
Il-17a	TTTAACTCCCTTGGCGCAAAA	CTTTCCCTCCGCATTGACAC	Amir Kumar Sigh, Nature Communications, 2018
TNFα	GACCCTCACACTCAGATCATCTTCT	CCACTTGGTGGTTTGCTACGA	Eric Féraille, PLoS One, 2014
IL-22	CATGCAGGAGGTGGTGCCTT	CAGACGCAAGCATTTCTCAG	Tomkovich, Cancer Res, 2017

### DNA extraction and 16S DNA sequencing

DNA was extracted using the protocol of Godon et al.^75^. DNA concentration and purity were determined using a NanoDrop instrument. We used the KAPA HiFi HotStart ReadyMix with 10 ng of extracted DNA for PCR amplification of the V3–V4 region of 16S rDNA. The amplification was performed with the primers MSQ-16SV3F (CTTTCCCTACACGACGCTCTTCCGATCTACGGRAGGCWGCAG) and MSQ-16SV4R (GGAGTTCAGACGTGTGCTCTTCCGATCTTACCAGGGTATCTAATCCT). PCR conditions were: 95 °C for 3 min; 30 cycles at 98 °C for 20 s, 65 °C for 30 s, and 72 °C for 1 min; and a final extension of 72 °C for 10 min. Amplification products were checked via electrophoresis on 2% agarose gel. Band size was approximately 500 bp. Sequencing was performed at the @BRIDGe facility at INRA Jouy-en-Josas using the Illumina Miseq (250 × 2 bp) system.

### Statistical analysis of sequencing data

16S sequencing data were uploaded to the Galaxy platform^76,77^ at https://galaxy.migale.inra.fr/. We used the FROGS pipeline to produce abundance tables of operational taxonomic units (OTUs). Denoising and clustering were performed with SWARM, chimera removal with VSEARCH, and affiliation with RDP Classifier using the 16S_SILVA_Pintail100_138 database. OTUs with a minimum presence of 0.005% in all sequences were kept. OTUs were filtered for a minimum identity of 97% and a minimum coverage of 95%. Measures were calculated of α- (observed and Shannon index) and β-diversity (Bray–Curtis dissimilarity score). Analysis of differential expression was performed using RStudio version 3.6.1 with the DESeq2 package.

### Statistical analysis of macroscopic symptoms and inflammatory markers

Graphics were created in Prism-GraphPad. Macroscopic results are represented as mean ± standard deviation (SD). Data was checked for normal distribution with the Shapiro–Wilk test. Outliers were removed from normally distributed data using the ROUT method (Q = 1%). All histograms were analyzed using non-parametric one-way ANOVA (Kruskal–Wallis) with Dunn’s post-hoc test. ELISA and qPCR results were represented in Tukey boxplots. Two-way analysis of variance (ANOVA) with Turkey’s post-hoc test was performed on weight or DAI progression data if no values were missing due to protocol interruption. If there were missing values, two-way ANOVA with Dunnett's multi-comparison post-hoc test was performed. In all figures, *P < 0.05, **P < 0.01, and ***P < 0.001.

### Ethics approval

This study was carried out in accordance with the guidelines of the local ethics committee.

## Supplementary Information

Supplementary Figures.

Supplementary Tables.

## Data Availability

https://data.inrae.fr/dataset.xhtml?persistentId=doi:10.15454/CJ26OO.
