# Supplementary material for: Lactococcus lactis engineered to deliver hCAP18 cDNA alleviates DNBS-induced colitis in C57BL/6 mice by promoting IL17A and IL10 cytokine expression
Source: Sci Rep. 2022 Sep 19;12:15641. doi: 10.1038/s41598-022-19455-3 (PMC9485145; doi:10.1038/s41598-022-19455-3)
Supplement: Supplementary file 1 — Supplementary Figures. [file 41598_2022_19455_MOESM1_ESM.pptx]

## Slide 1
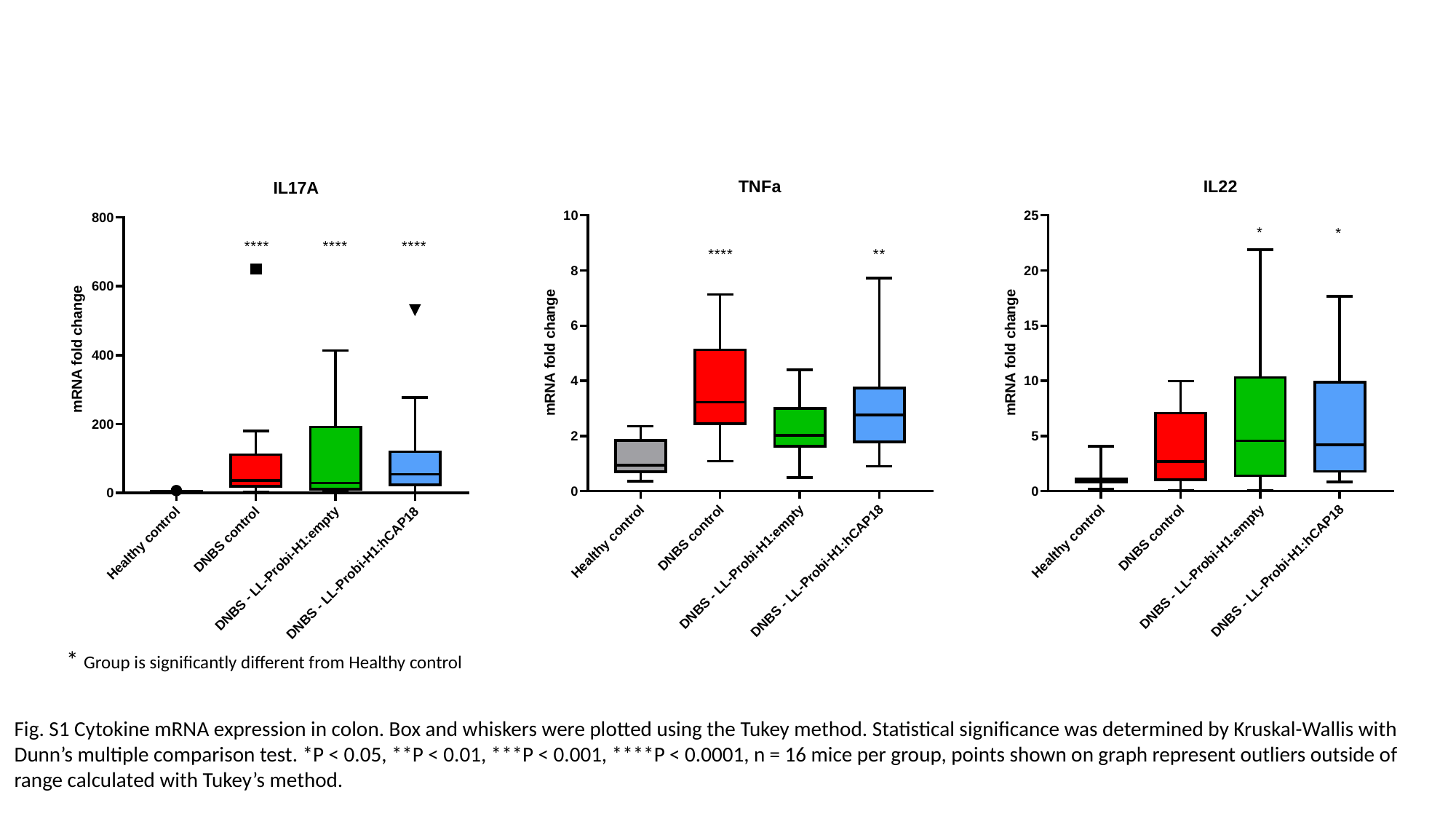

* Group is significantly different from Healthy control
Fig. S1 Cytokine mRNA expression in colon. Box and whiskers were plotted using the Tukey method. Statistical significance was determined by Kruskal-Wallis with Dunn’s multiple comparison test. *P < 0.05, **P < 0.01, ***P < 0.001, ****P < 0.0001, n = 16 mice per group, points shown on graph represent outliers outside of range calculated with Tukey’s method.

## Slide 2
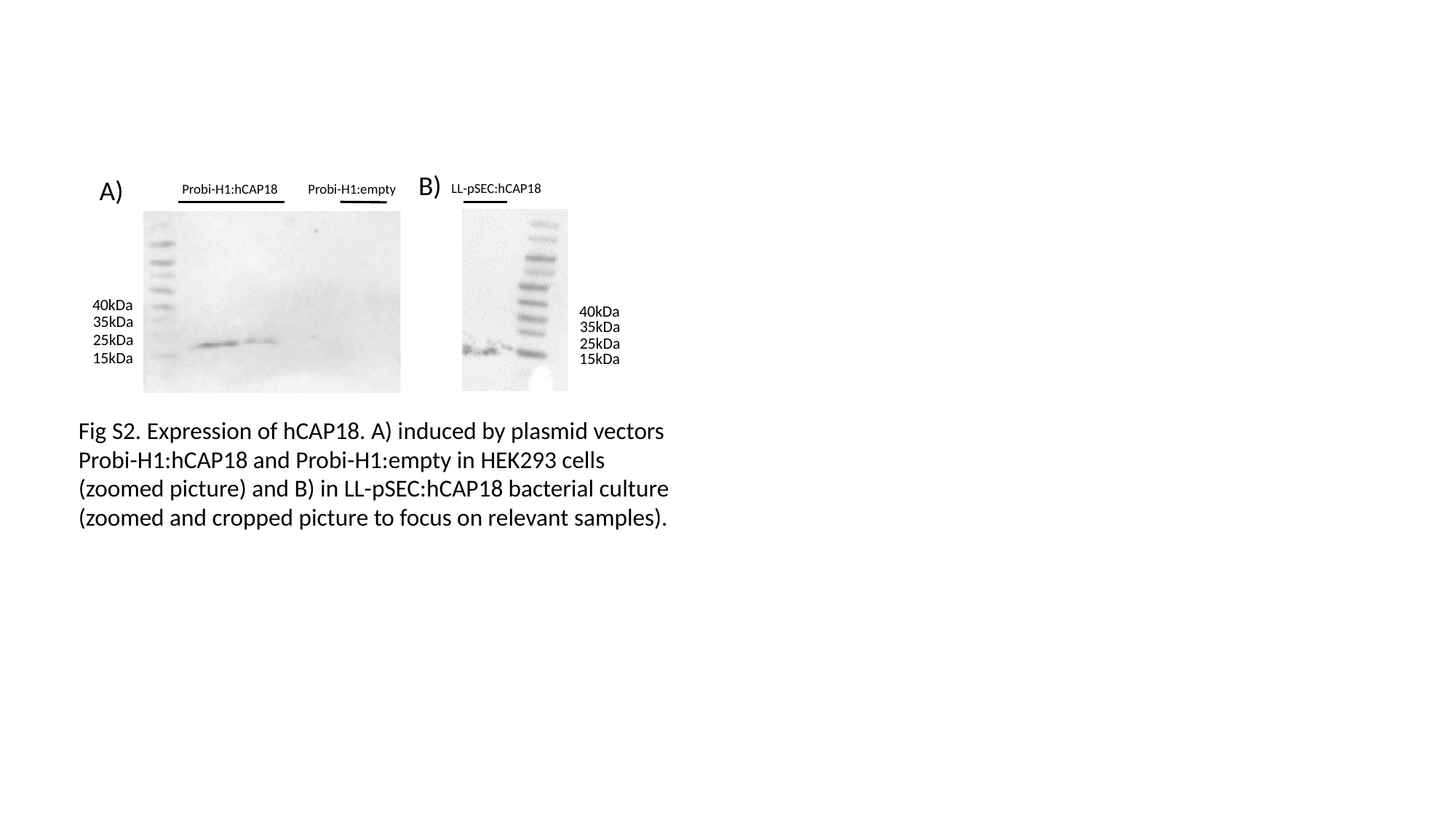

B)
A)
LL-pSEC:hCAP18
Probi-H1:hCAP18
Probi-H1:empty
40kDa
40kDa
35kDa
35kDa
25kDa
25kDa
15kDa
15kDa
Fig S2. Expression of hCAP18. A) induced by plasmid vectors Probi-H1:hCAP18 and Probi-H1:empty in HEK293 cells (zoomed picture) and B) in LL-pSEC:hCAP18 bacterial culture (zoomed and cropped picture to focus on relevant samples).
