# Supplementary material for: Lactococcus lactis engineered to deliver hCAP18 cDNA alleviates DNBS-induced colitis in C57BL/6 mice by promoting IL17A and IL10 cytokine expression
Source: Sci Rep. 2022 Sep 19;12:15641. doi: 10.1038/s41598-022-19455-3 (PMC9485145; doi:10.1038/s41598-022-19455-3)
Supplement: Supplementary file 2 — Supplementary Tables. [file 41598_2022_19455_MOESM2_ESM.docx]

**Table S1: PERMANOVA of samples by treatment**

|  | **Df** | **SumsOfSqs** | **MeanSqs** | **F.Model** | **R2** | **Pr(>F)** |
| --- | --- | --- | --- | --- | --- | --- |
| treatment | 2 | 0.25595 | 0.127974 | 1.8079 | 0.11437 | 0.005 ** |
| Residuals | 28 | 1.98198 | 0.070785 |  | 0.88563 |  |
| Total | 30 | 2.23793 | |  | 1.00000 |  |

**Table S2: PERMANOVA of samples by macroscopic score**

|  | **Df** | **SumsOfSqs** | **MeanSqs** | **F.Model** | **R2** | **Pr(>F)** |
| --- | --- | --- | --- | --- | --- | --- |
| macroscopic_score | 5 | 0.37771 | 0.075542 | 1.0152 | 0.16878 | 0.415 |
| Residuals | 25 | 1.86022 | 0.074409 |  | 0.83122 |  |
| Total | 30 | 2.23793 |  |  | 1.00000 |  |

**Table S3: Differential abundance results contrasting No treatment, LL-probi-H1:hCAP and LL-probi-H1:empty mice**

| No treatment/LL-probi-H1:LL37 | | | | | |
| --- | --- | --- | --- | --- | --- |
|  | **Cluster** | **Level** | **log2foldchange** | **p-value** |  |
| *Muribaculaceae* | Cluster_116 | Family | 1.32404293589324 | 1.6570409959217e-06 | |
| *Muribaculaceae* | Cluster_55 | Family | 1.14388973387858 | 3.40234461679945e-06 | |
| *Parasutterella* | Cluster_11 | Genus | 1.4503773082355 | 5.2549085927291e-05 | |
| *Peptostreptococcaceae* | Cluster_141 | Family | 2.66339496154198 | 9.38065654201224e-05 | |
| *Clostridia UCG-014* | Cluster_158 | Order | 1.75387106026085 | 0.000151874773353639 | |
| *Ruminococcaceae UCG-010* | Cluster_475 | Family | 2.29449509395752 | 0.00019590949751841 | |
| *Muribaculaceae* | Cluster_38 | Family | 0.857144634741257 | 0.00043322197031411 | |
| *Muribaculaceae* | Cluster_232 | Family | 1.20920112936848 | 0.00159431331890297 | |
| *Muribaculaceae* | Cluster_661 | Family | 1.17721968770559 | 0.00139246648114039 | |
| *Lachnospiraceae NK4A136 group* | Cluster_36 | Genus | -4.64021961088753 | 0.0017318763725779 | |
| *Ruminococcaceae UBA1819* | Cluster_70 | Genus | -2.00596554250594 | 0.00196514952988505 | |
| *Oscillibacter* | Cluster_273 | Genus | -1.58415720227679 | 0.00153927737844786 | |
| *Clostridium sensu stricto 1* | Cluster_47 | Genus | 2.38624221560097 | 0.00197974019783422 | |
|  |  |  |  |  |  |
| No treatment/LL-probi-H1:empty | | | | | |
|  | **Cluster** | **Level** | **log2foldchange** | **p-value** |  |
| *Lachnospiraceae NK4A136 group* | Cluster_139 | Genus | -4.30165777892058 | 1.31001136762978e-05 | |
| *Lachnospiraceae bacterium 595* | Cluster_255 | Species | -5.52516890734503 | 1.87369438854964e-05 | |
| *Muribaculaceae* | Cluster_116 | Family | 1.06318828641114 | 8.75680777001193e-05 | |
| *Clostridia UCG-014* | Cluster_49 | Order | 2.43677858700927 | 0.000109045286356505 | |
| *Ruminococcaceae UCG-010* | Cluster_845 | Family | 3.30039065167253 | 0.000279936091093825 | |
| *Lachnospiraceae NK4A136 group* | Cluster_944 | Genus | -4.60791171917626 | 0.000421624171382098 | |
